# Influence of Holes Manufacture Technology on Perforated Plate Aerodynamics

**DOI:** 10.3390/ma14216624

**Published:** 2021-11-03

**Authors:** Joanna Grzelak, Ryszard Szwaba

**Affiliations:** 1 Faculty of Mechanical Engineering and Ship Technology, Gdansk University of Technology, 11/12 Narutowicza, 80-233 Gdansk, Poland; 2 Institute of Fluid Flow Machinery, Polish Academy of Sciences, Fiszera 14, 80-231 Gdansk, Poland; rssz@imp.gda.pl

**Keywords:** perforated plates, laser micromachining, holes manufacture, flow direction

## Abstract

Transpiration flow is a very important and still open subject in many technical applications. Perforated walls are useful for the purpose of “flow control”, as well as for the cooling of walls and blades (effusive cooling) in gas turbines. We are still not able to include large numbers of holes in the numerical calculations and therefore we need physical models. Problems are related also to the quality of the holes in perforated plates. The present transpiration analysis concerns with experimental investigations of the air flow through perforated plates with microholes of 125 and 300 µm diameters. A good accordance of the results with other experiments, simulations and theory was obtained. The received results very clearly show that technology manufacturing of plate holes influences on their aerodynamic characteristics. It turned out that the quality of the plate microholes using laser technology and, consequently, the shape of the hole, can affect the flow losses. Therefore, this effect was investigated and the flow characteristics in both directions were measured, i.e., for two plate settings.

## 1. Introduction

Perforated plates have been used for many years in numerous industrial sectors and have been the subject of intensive research for decades [1,2,3]. Areas of application range are very different, from absorption of sound [4], separators and micro filters [5,6], heat exchangers [7,8], to a method of homogeneous flow generation [9] and a new cooling concept for blade turbines [10]. Transpiration flows are also used in flow steering devices to control the boundary layer separation, as well in passive shock wave control [11,12]. Laminar technology, which uses suction with perforated plates, is worth mentioning too. These aspects have been presented in works on laminar wing technology [12,13]. Perforated plates are the basis for microchannel cooling (or effusive cooling) of blades of gas turbines, where aerodynamics and heat transfer interaction take place. In this case, the use of plates allows the coolant to be distributed in a cooling layer with much better uniformity, as well as better transport of the coolant to the blade surface and creating better protection of the blade surface from hot gases [14].

The above examples of perforated plate applications, however, require the hole sizes in the order of 100 µm. Even a small area with a perforation of approximately 5% already requires thousands of holes. In this case, the use of drilling technology is unlikely to be an option due to the high costs involved. Additive manufacturing (3D printing) is also not a solution either, because with these hole sizes the powder material cakes inside the hole. The only technology which can cope with making such a large number of holes is laser technology.

The laser micromachining is most often carried out through direct illumination of the workpiece by the focused laser beam (so-called “direct writing”) [15]. The lasers can operate in continuous-wave mode and also in pulsed mode. Pulse length for both lasers modes can be adjusted from 10 μs and 50 ms, with the exact range depending on the parameters used and laser model. During micromachining the laser pulses are focused on the material surface (e.g., metal). The absorbed energy is transported deep into the material in the process of thermal conductivity. The material located in the so-called heat-affected zone (HAZ) partially melts and then evaporates. However, it usually comes with the degradation of the processed material as a result of its damage around the micromachining spot. The consequence of such process are thermal effects as, e.g., deformation, sintering or discoloration of the edge of the material [16,17]. The disadvantages of laser technique include relatively low accuracy (on the level of several micrometers), and possibility of thermal damage of the workpiece material in HAZ.

Transpiration flows can be modeled in several ways. The first one that does not take into account the flow losses through the microholes is the isentropic model. Models supported by experimental data have been proposed, for example, in the works of Poll [18] or Inger and Babinsky [19,20]. They all relate to the air flow through cylindrical holes and include constants, which take into account the imperfections of the microholes. The models proposed by Inger and Babinsky contain the influence of viscosity and compressibility and also the shape of the opening of the hole. Numerical modeling of anisotropic drag for a perforated plate with an array of cylindrical holes was carried out in Youngmin and Young [21], but these results cannot be used for technical issues.

In experimental studies on perforated plates, it is usual to determine the characteristics of such a plate, i.e., the dependence of the pressure drop across the plate as a function of the mass flow rate. Such formulas are then successfully applied in numerical simulations [22]. The problem in experiments using perforated plates is less important that the hole channel performed by the laser is not actually cylindrical, as this feature can be taken into account in aerodynamic perforation. With hole sizes in the order of hundreds of micrometers, a serious problem from an aerodynamic point of view is that the hole diameter made on the laser side is slightly different from that on the exit side, causing the hole channel to become conical. This channel shape will affect the aerodynamic characteristics depending on which diameter, larger or smaller, is at the inlet of the channel. Therefore, the motivation, the aim for this work, was to show the influence of micro-hole technology on the aerodynamic characteristics of perforated plates. When using such plates in various applications, one should keep in mind that a given characteristic corresponds to a specific flow direction. With a change in the direction of the flow in the holes of a given plate, its characteristics change, as will be shown below in the experimental studies.

## 2. Experimental Setup

This paper concerns the flow direction effects across the perforated plates on their aerodynamic characteristics. The study of plate aerodynamic characteristics with micro-holes was carried out using general experimental data on a macro scale. In this regard, experimental investigations were performed using air as medium over two perforated plates with parameters displayed in Table 1, where D is a hole diameter, L—hole channel length, S—perforation, F—plate area.

Investigations have been performed for a range of Mach numbers from *Ma* < 0.1 up to choked condition of the flow in microholes, *M* = 1. The flow of compressible gas dynamics in channels has been studied considering different aspects of flow physics and can be found in various books and papers, e.g., the continuum approach [23], the molecular approach [24] and general analytical relations [25]. The basic theory necessary to obtain information from experimental data is outlined below. The equation of state of gas represents the relationship between the parameters of an ideal gas. When this equation is transformed, the speed of sound is defined as:

(1) $$a=\sqrt{\kappa RT}=\sqrt{\frac{\kappa p}{\rho }}$$

*R*—gas constant, *κ*—specific heat constant, *p*—pressure, *T*—temperature, *ρ*—density.

Molecular properties related to the size of the continuum is another characteristic parameter of gas, namely the mean free path between the gas molecules translates into the average distance between them and is described as:

(2) $$\lambda =\frac{k_{\lambda }\mu (T)\sqrt{2RT}}{p}$$

where $k_{\lambda }=\frac{\sqrt{\pi }}{2}$. Viscosity in this equation depends on the temperature and could be defined as:

(3) $$\mu (T)={\left(\frac{T}{T_{ref}}\right)}^{\varpi }$$

*k*—thermal conductivity, *μ*—dynamic viscosity, *p*—pressure, *ω*—viscosity index, *U*— velocity.

The dimensionless parameters that determine the appropriate scales in the flows are defined by the Reynolds number *Re* and the Mach number *Ma*. The characteristic quantities which determine these parameters are based on the hole diameter and the average flow velocity, i.e., $Ma=\frac{U}{a}$, $Re=\frac{\rho UD}{\mu }$.

**Figure 1:**
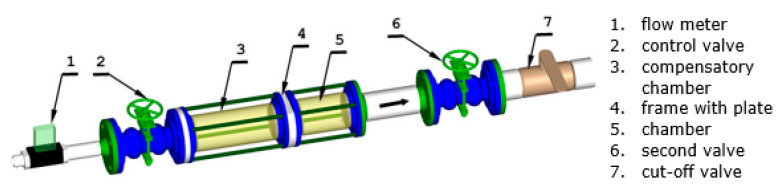
The test section view.

**Figure 2:**
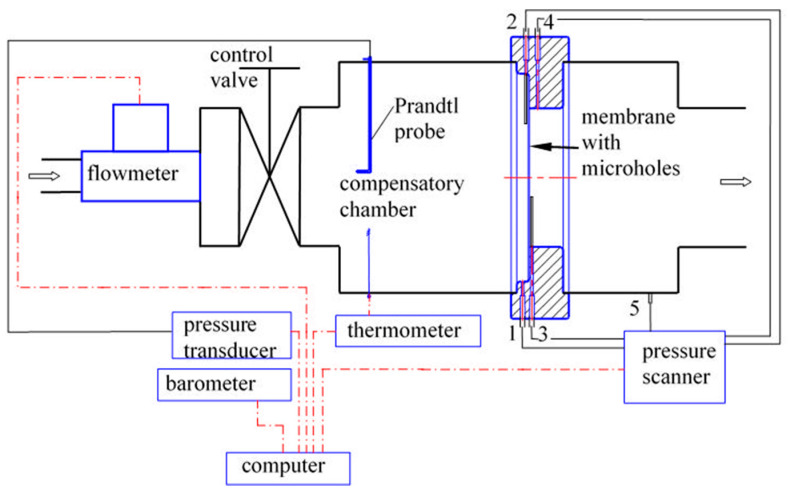
Arrangement of the measurement system.

Experiments were conducted in the measurement section which is shown in Figure 1. The arrow in the figure indicates the direction of the flow, which is from left to right. The flow resulting from the pressure variance between the ambient and the pressure in the vacuum vessels downstream of the Valve 7. The air parameters in the laboratory space, i.e., the temperature of approximately 20–22 °C and the atmospheric pressure on a given measurement day correspond to the ambient conditions. The air flow starts from the ambient through the Flow Meter 1, further through the Control Valve 2, Compensation Chamber 3 to Frame 4. The tested perforated plate with micro holes is mounted in Frame 4. Behind the plate, air flows through Chamber 5, Valve 6 (the flow condition controller behind the micro-holes) and follows through cut-off Valve 7 into the vacuum vessels. A schematic diagram of the measurement system is shown in Figure 2.

During the experimental campaign, the following quantities were measured: mass flow rate, pressures, and temperature at the relevant points. Since the mass flow rate varied significantly depending on the pressure difference on both sides of the perforated plate, therefore depending on the mass flow range, this value was measured by means of three various laminar flow meters, (Alicate: 20, 100, 1500; Tucson, AZ, USA; SLPM; Standard Litre Per Minute at ambient condition, i.e., pressure of 1013 hPa and temperature of 298 K). The accuracy of mass flow rate measurement is ±0.01 SLPM of measured value. The stagnation parameters, i.e., pressure and temperature were measured in Compensation Chamber 3. Pressure was measured using a Dwyer Prandtl probe (Dwyer, Michigan City, ND, USA), Model 167-6, with a diameter of 1/8”. A Kulite pressure transducer (Kulite, Leonia, NJ, USA) with accuracy of 0.1% (FS, full scale), i.e., ±1 hPa, was connected to this probe. Temperature was measured using a thermoelement (Czaki, Raszyn, Poland) with accuracy of 0.1 K. The pressure before and after the perforated plates was measured using a pressure scanner (MEAS, Hampton, VA, USA) with accuracy of 0.05% (FS), i.e., ±0.5 hPa.

The investigations of the flow through a porous plate were carried out under ambient conditions. The specified mass flow rate flows over area *F*, which induces a pressure difference Δ*P* = *P*_in_ − *P*_out_. The pressure *P*_in_ was equal to the atmospheric pressure *P*_0_ and the pressure *P*_out_ downstream the plate was set using Valve 6. In this way, with appropriate manipulation of the control valve, the individual characteristics can be measured.

**Figure 3:**
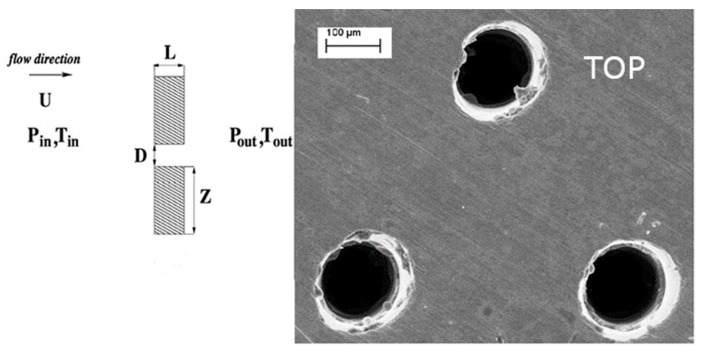
Schematic illustration of the perforated plate and the microscopic picture.

A detailed zoomed view of the plate is shown in Figure 3. The diameter of perforated plates was 60 mm and they were manufactured from stainless steel. A microscopic view of K1 plate is shown in Figure 3. This is the view on the inlet side respect to the laser position and one can notice the degradation of the holes edge as a result of its damage around the laser spot. The accuracy of micro-hole diameter assessment is 10 μm and its width is 2 μm. The perforation values were given by the producer of plates. These values are presented in Table 1. The production porosity specification of plates K1 and K3 was a 4.1 and 5.7%, respectively. Actually, the aerodynamic porosity (the value that is measured during experiments) differs from that declared by the producer.

Aerodynamic porosity informs about what mass rate actually flows through the plate with a given cross-section (F) and at a given pressure difference. The differences between geometric and aerodynamic porosity are due to the inaccuracy of the hole manufacture and gas-dynamic effects. Hence, we use these two different definitions of the porosity. The porosity declared by the manufacturer results from the settings of the technological process, i.e., the diameter of the holes and their arrangement on a given surface. Aerodynamic porosity considers all these imperfections. The exact way of determining the aerodynamic porosity is presented in [26,27].

**Figure 4:**
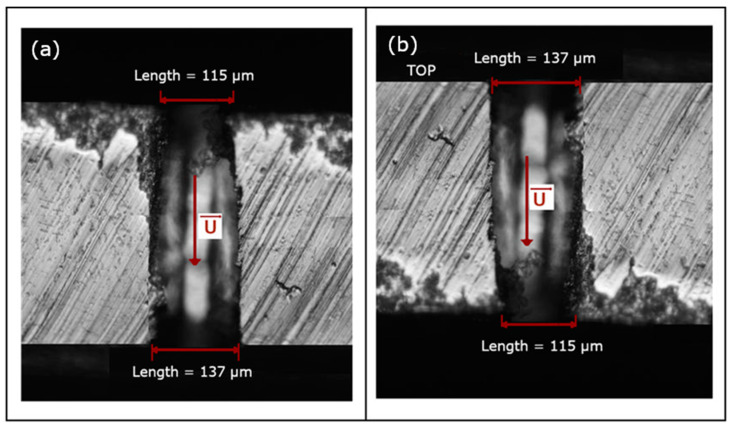
Shape of the hole channel with the velocity direction for the basic (**a**) and reversed (**b**) plate setting; plate K1.

To verify the influence of the hole channel shape on the aerodynamic characteristics, the given plate was placed with the smaller hole opening at the inlet first (Figure 4a) and then reversed to obtain the bigger hole opening at the inlet (Figure 4b). The results for the latter plate setting are named as ‘reversed’ (rev). A 137 μm-diameter hole drilled in the plate represents the entrance of the laser beam (marked as TOP in Figure 3 and Figure 4b), while 115 μm-diameter is the exit. The exit of the laser beam characterises with the smaller diameter and the conical shape of the hole. The lengths of the holes diameter were calculated by the computer program (Dynamic Studio 4.4.3). The quality of the picture, resolution, lighting, and contrast seen under the microscope is incomparably better compared to what can be seen in the picture in the article, so the measurement error is estimated at approx. ±2 px, which corresponds to approx. ±1 μm.

Measurement uncertainties were estimated for the mass flow rate and the pressure difference, but they are very small and almost not visible on the picture (they are smaller than the chart points). To calculate the uncertainties of the above-mentioned quantities, exact differentials were used, containing such variables as pressure, temperature and air density. Average value of the obtained measurement errors for the mass flow rate are equal to 1.7%. High-accuracy pressure transducers (MEAS, Hampton, VA, USA) were used to measure the pressure, therefore the measurement errors for the pressure difference were smaller than 0.04%.

## 3. Experimental Results

**Figure 5:**
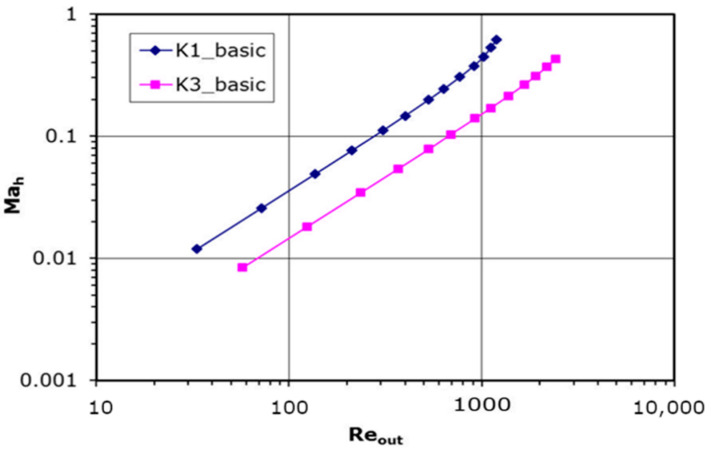
Mach number versus Reynolds number plot.

The results presented in this section correspond to the basic plate setting (Figure 4a). The Reynolds number *Re_out_* is based on the orifice’s diameter and outlet parameters. The Mach number in the plate hole, *Ma_h_*, is Reynolds numbers function (Figure 5). As can be noted, except for one point for K1, where *Re* = 33, there can be distinguished only one flow regime for Reynolds numbers higher than 50, which is linked to the turbulence transition. It can be also noticed the effects of compressibility in flow through the holes of perforated plate. The flow velocity (*Ma_h_*—non-dimensional velocity coefficient) distribution at a high Reynolds number changes its linear character. These effects are stronger for the smaller hole diameter of the perforated plate.

The mass flow rate *Q* is specified by the formula from the experimental studies in Reference [26]:

*Q* = *FSϱ_h_U_h_* (4)

**Figure 6:**
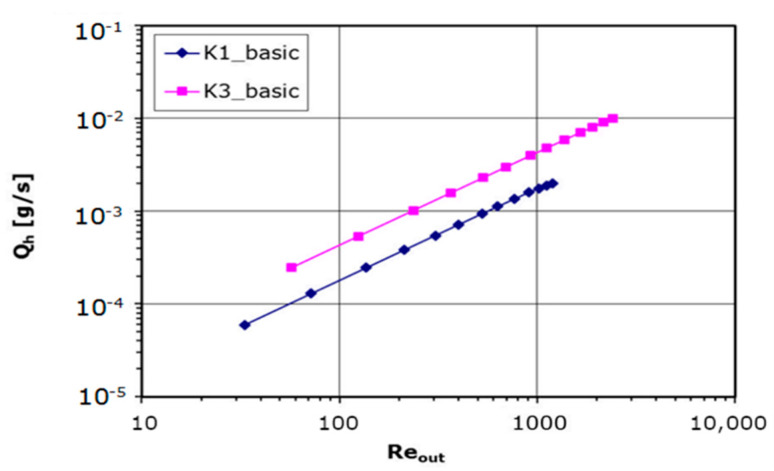
Mass flow rate versus Reynolds number plot.

The mass flow rate for a single hole, *Q_h_* = *Q*/*N*, as a function of Reynolds number is shown in Figure 6. Since the velocity resulting from the mass flow rate is a variable in the calculation of Reynolds number, the nature of this relationship is not unexpected. Therefore, the dependency between the mass flow rate *Q_h_* and the Reynolds number is saw as almost linear (small deviation for high Reynolds numbers) and plotted in Figure 6. Higher mass flow rates for the same Reynolds number are obtained for the larger holes orifice in the whole range of the parameters.

### 3.1. Aerodynamics of Perforated Plates

The aerodynamics of perforated plates which was studied for years in many different experiments has been focused on plates with large holes. Examples that can be found in the literature concern for example the pressure loss coefficient over thickness of plate [28], turbulent flow over the perforated plate [9] and high Reynolds numbers (*Re* > 10,000) [29]. In this paragraph, the obtained results will be compared with the ones of two specific paper that considers various hole diameters in different kinds of plates [26,27]. Implementing the stagnation parameters to Equation (4), we obtain Equation (5).

(5) $$Q=FS\frac{Ma_{h}}{{\left(1+\frac{\kappa -1}{2}Ma_{h}\right)}^{\frac{\kappa +1}{2(\kappa -1)}}}P_{0}\sqrt{\frac{\kappa }{RT_{0}}}$$

The experimental formula obtained in Reference [18] leads to:

(6) $$Ma_{h}=1.2\;{\left(\frac{\Delta P}{P_{0}}\right)}^{0.55}$$

**Figure 7:**
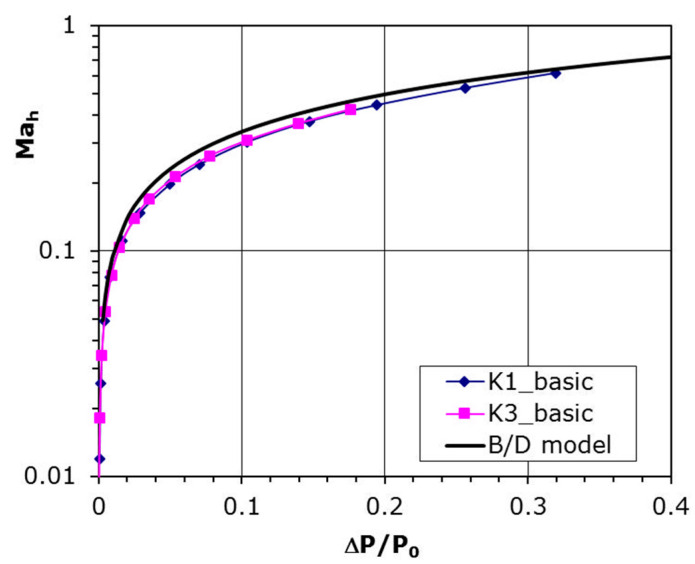
Comparison with other experiment for perforated plates in parallel flow, B/D model.

The curve resulting from Equation (6) is plotted together with experimental data in Figure 7. This plot shows that velocity coefficient is tending to unity with a pressure drop on both sides of the plate increases. The experimental data coincide very well with that model and the precise fitting coefficients are introduced in Table 2 beneath, where that relation is specified by the formula:

(7) $$Ma_{h}=A\;{\left(\frac{\Delta P}{P_{0}}\right)}^{B}$$

It can be seen that Coefficients A and B are very similar to that from Equation (6) and quite good compatibility with B/D model is achieved.

### 3.2. Characteristics Depending on the Flow Direction

**Figure 8:**
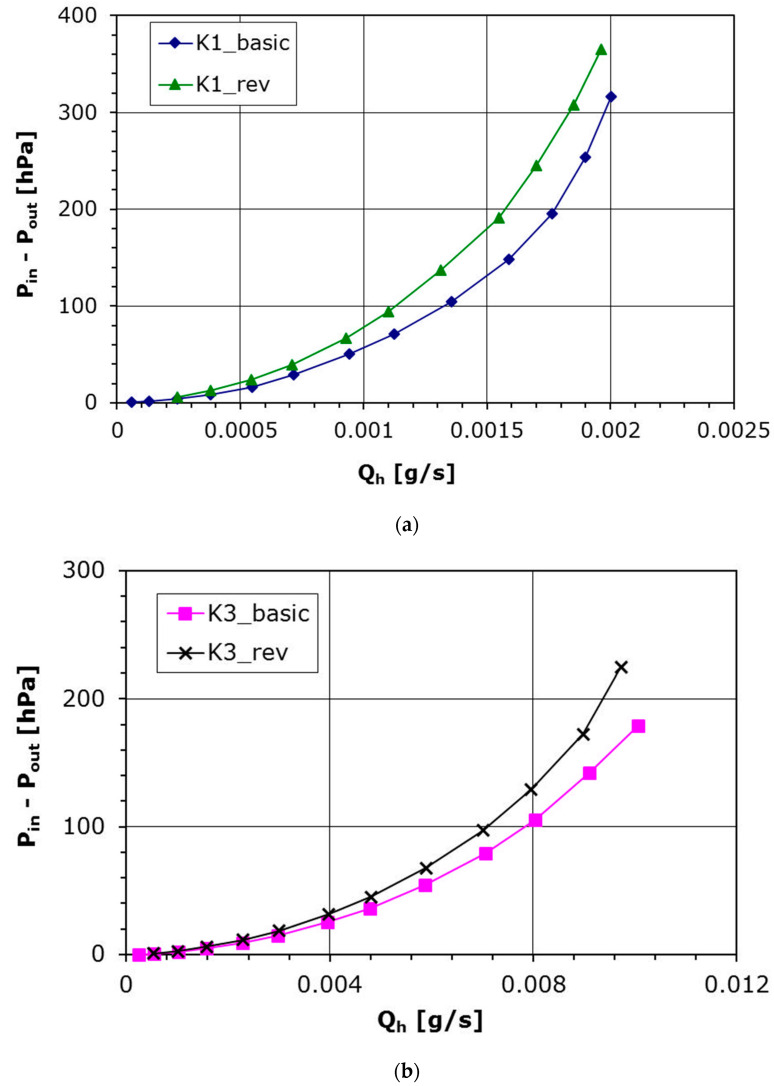
(**a**) Pressure difference as a function of the mass flow rate for basic and revised plate setting; Plate K1. (**b**) Pressure difference as a function of the mass flow rate for basic and revised plate setting; Plate K3.

**Figure 9:**
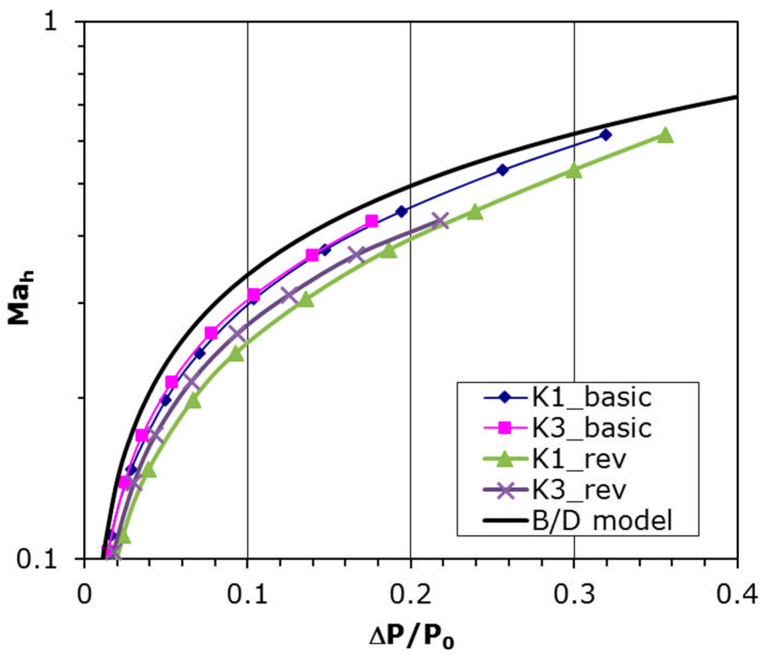
Comparison with B/D model for both plate settings and Plates K1, and K3.

Analysis connected with perforated plates usually involve cylindrical hole assumption, which is true when holes are made using drilling technology. In this experiment, laser technology was used to make the perforated plates, hence the hole channel has a shape closer to conical than to cylindrical. To verify if the shape of the hole channel really influences the aerodynamic characteristics, the given plate has been reversed to set the bigger hole opening at the inlet. The results for such plate setting are shown in Figure 8a (plate K1) and Figure 8b (plate K3). The pressure difference is a function of the mass flow rate in the hole, *Q*_h_. It can be noted that for the same pressure difference the mass flow rate is smaller in the case of ‘reversed’ setting for both plates with various diameter. It is also seen that the Coefficients *A* and *B* from Equation (7) for the latter case also differ to those for the basic plate setting (Table 3 and Figure 9). The velocity in the hole increases slower with the pressure drop, which indicates a higher aerodynamic drag in the flow for the ‘reversed’ plate setting.

The above results show how much the manufacturing of plate holes influences the aerodynamic characteristics. A lower pressure drop at the same flow rate was obtained for the basic setting, i.e., the smaller hole cross-section is located at the inlet of the flow through the plate. This behaviour is related to the shape of the hole, i.e., in subsonic flow with increasing cross-sectional area the flow velocity decreases and the pressure in the hole increases, which translates into a lower pressure drop across the plate compared to the reverse setting where the flow in the hole accelerates and the pressure loss increases.

The above results show how important the hole performance technology is in relation to the aerodynamic characteristics of perforated plates. If the laser technology is chosen, due to its influence on the shape of the holes as described in detail above, it is necessary before using perforated plates to accurately determine the direction of flow in the conical hole in the flow characteristics study of the plate. Further on, in the specific application of perforated plates, it is imperative that this flow direction is respected, i.e., the correct positioning of the plate in direction of the pressure drop in the flow.

## 4. Conclusions

This paper concerns the laser technology effects on the flow across the perforated plates and their aerodynamic characteristics. The measurements of the aerodynamic performance of the plate with microholes were carried out using the universal macroscale experimental data. Aerodynamic characteristics for two different plates with different perforations, hole diameters and channel lengths were presented. To verify the influence of the hole manufacture technology on the plates characteristics, two flow directions (basic and reversed plate setting) were studied.

The investigation showed that the laser technology of making microholes is not indifferent to the flow direction through the plate. For the same pressure difference the mass flow rate is smaller in case of ‘reversed’ setting for both plates, i.e., the smaller hole cross-section is located at the outlet of the flow through the plate. The experimental data were also compared with Equation (6) representing the B/D model. A good accordance was obtained, although the Coefficients *A* and *B* from Equation (7) are different for different plate settings. The received results very clearly show the technology manufacturing of plate holes influences on their aerodynamic characteristics. Perhaps a larger range of parameters (diameter, perforation) would show a greater difference. Nevertheless, this investigation requires continuation in the future.

In the case of perforated plate applications where the required holes diameter in the plate is below 0.3 mm, there is practically no choice of hole manufacturing technology, and we are forced to choose the laser technology. It is, therefore, very important that before using perforated panels, it is necessary to accurately determine the flow direction in the conical shape hole in the flow characteristics study of the plate, i.e., the same position of the plate in the direction of the pressure drop across the perforated plate both in the investigation and in the application.

## Figures and Tables

**Table 1:** Plates parameters in the experiment.

Plate Name	D (mm)	L/D	S (%)	F (m^2^)
K1	0.125	8.00	4.09	0.00283
K3	0.3	4.67	5.70	0.00283

**Table 2:** Coefficients for the Equation (7) formula, Mach number dependence from pressure drop.

Plate	*A*	*B*
K1_basic	1.22	0.60
K3_basic	1.21	0.59

**Table 3:** Coefficients for the Mach number pressure drop dependency for ‘revised’ plate setting.

Plate	*A*	*B*
K1_rev	1.06	0.60
K3_rev	1.04	0.58

## Data Availability

The data supporting the reported results of this study can be made available from the corresponding author, upon request.
